# CBT4CBT web-based add-on treatment for cocaine use disorder: Study protocol for a randomized controlled trial

**DOI:** 10.3389/fpsyt.2023.1051528

**Published:** 2023-03-02

**Authors:** Núria Mallorquí-Bagué, Alba Palazón-Llecha, Mercè Madre, Francesca Batlle, Santiago Duran-Sindreu, Joan Trujols

**Affiliations:** ^1^Addictive Behaviors Unit, Department of Psychiatry, Biomedical Research Institute Sant Pau (IIB Sant Pau), Hospital de la Santa Creu i Sant Pau, Barcelona, Spain; ^2^Department of Psychology, University of Girona, Girona, Spain; ^3^Centro de Investigación Biomédica en Red de Salud Mental (CIBERSAM), Instituto de Salud Carlos III (ISCIII), Madrid, Spain

**Keywords:** cocaine use disorder, treatment, cognitive behavioral therapy, treatment outcome, gender, predictive factors, clinical trial, protocol

## Abstract

**Background:**

Cocaine use disorder (CUD) is a chronic condition that presents high relapse rates and treatment dropouts. Web-based interventions have proven to be effective when optimizing face-to-face treatments in different mental health conditions and have the potential to optimize current CUD treatments. However, web-based interventions in addictive behaviors are still limited. The aim of this study is to evaluate whether adding a web-based cognitive behavioral therapy (i.e., CBT4CBT) to standard CUD treatment, improves treatment outcomes in a Spanish sample of patients with severe CUD (which requires inpatient treatment). Additionally, we aim to explore predictive factors of treatment response and treatment gender-related differences.

**Methods:**

All individuals coming for inpatient cocaine detoxification who meet the inclusion criteria will have the possibility to be part of the study. The participants of this open-label randomized controlled clinical trial (RCT) will be allocated to treatment as usual (TAU) or TAU+CBT4CBT after the hospitalization for cocaine detoxification. During the inpatient treatment they will all receive an individualized psychological intervention. There will be six time point assessments: at 48–72 h of starting inpatient treatment, at the end of inpatient treatment and before starting day care and outpatient treatment, at the end of the 8 weeks CTB4CBT / TAU arm treatment and at three follow-up time points (1-, 3-, and 6-months post-treatment).

**Discussion:**

To the best of our knowledge, this is the first RCT that explores the efficacy of adding a web-based cognitive behavioral therapy to usual CUD treatment with patients of a clinical sample in Europe.

**Trial registration:**

IIBSP-CTB-2020-116, NCT05207228. Submitted 8^th^ of April 2021, posted 26 ^st^ of January 2022. https://clinicaltrials.gov/ct2/show/NCT05207228?cond=Cocaine+Use+Disorder&draw=2&rank=1.

## Introduction

Cocaine use disorder (CUD) is a complex condition with great impact and burden for patients, their relatives and society (1, 2). Due to its increasing incidence rate in Europe and US during the last decade (2, 3) and to its highly prevalent chronic course (4), where dropout rates are high and remission is often followed by relapse, there is a large and increasing need to improve current CUD treatments. Thus, far, no pharmacological approaches are yet approved for treating CUD, which often is a resistant-to-treat condition and is associated to significative morbidity and mortality rates (5, 6). Clinical evidence-based guidelines recommend psychosocial interventions as first line of treatment (1, 2) with large body of scientific knowledge suggesting that these interventions can help improve CUD; especially, contingency management (CM) and cognitive-behavioral therapy (CBT) (1, 5–8).

Systematic reviews and meta-analysis testing the effectiveness of the available psychosocial interventions for individuals with CUD, converge in reporting the efficacy of CM programs either alone (7) or in combination with community reinforcement approaches (CRA) or with other CBT approaches [such as the relapse prevention treatment developed by Carroll et al. (9–11)] by increasing the abstinence period and reducing the dropout rates, which makes CM the most reliable method to turn an active cocaine user into an abstainer (5, 6). CM and CRA are two behavioral approaches, based on the principle of operant conditioning, where the substance related behavior is understood to be reinforced or maintained by its consequences. The previous (CM), provides an arbitrary reward in face of positive behavioral changes (i.e., most frequently drug-negative urine test), which typically consists of vouchers exchangeable for foods and services, and the latter (CRA) focuses on providing natural rewards by helping people adopt a more rewarding lifestyle free of drugs. However, in clinical practice, the above mentioned approaches are not often implemented (12) in part because they require some cost-effective changes such as the need for conducting higher urine tests or providing monetary rewards such as vouchers, thus the approach of Carroll et al. (13, 14) is a good evidence-based alternative.

With the new digital era, a new intervention window emerges through the implementation of web-based treatments that can intensify and optimize the current face-to-face treatment approaches. In fact, the current challenge in the treatment of CUD is in identifying innovative ways to implement the treatment rather than developing more effective strategies (5). However, literature in clinical settings is still scarce for addictive behaviors with very few studies on the effectiveness of web-based interventions for the treatment of illegal substance use. Up until now, much of this work has been conducted in the area of smoking, in which cognitive-behavioral interventions by means of computers/ internet have demonstrated to be effective on quit rates or attempts (15, 16). Thus, far, two web-based interventions have been tested through RCTs for treating CUD with positive effects reported in their target population (17, 18).

CBT4CBT is a CBT web-based treatment for substance use disorders. Previous studies conducted in US have shown its positive impact on improving face-to-face treatment as usual (TAU) (18–20). More specifically, participants who underwent CBT4CBT were significantly more likely to attain three or more weeks of continuous abstinence during treatment compared to participants who underwent TAU (18–20). These effects endured 6 months after treatment, measured through self-reported measures and urine toxicology screens (18, 20). Additionally homework adherence in CBT4CBT is associated with less cocaine use during treatment (19) and participants who completed at least 50% of homework displayed a greater cocaine use reduction in comparison with the ones who completed <50% of homework (21). Considering previous data regarding the prognosis and standard CUD treatment approaches, the evidence suggests that CBT4CBT approach concomitant to TAU could enhance current treatments and improve the outcomes in patients with severe CUD. In order to test the add-on efficacy of the web based CBT4CBT program concomitant to TAU, the comparative and the experimental group will have the same protocol of face-to-face sessions and will only diverge with the provision of the web based CBT4CBT program at the experimental group.

### Aims of the study

The aim of the present study is to evaluate whether adding a web-based cognitive behavioral therapy (CBT4CBT) to standard cocaine use disorder treatment improves treatment outcomes in a Spanish sample of patients with severe CUD (that require inpatient treatment). To do so, two primary outcomes are considered: (1) treatment retention (vs. dropout) and, (2) relapse (number of positive urine specimens submitted, percent negative urine screens, percent days of abstinence, self-reported frequency of cocaine use). As secondary outcomes the following variables are measured: craving, psychopathological symptoms, days of any drug use, CBT4CBT usability and treatment satisfaction.

The secondary objectives of the study are two-fold: (a) To explore differences between men and women in treatment response; (b) To explore if comorbid psychopathological symptoms, other addictive behaviors, craving and emotion regulation difficulties are predictive factors of treatment outcome at the end of inpatient treatment as well as at the end of the 8-week CBT4CBT + TAU or TAU and at follow-ups (1, 3, and 6 month).

## Methods

### Participants

Inpatients with a diagnosis of cocaine use disorder (DSM-5 criteria) referred for cocaine detoxification to our addictive behaviors' unit at the Hospital de la Santa Creu i Sant Pau in Barcelona (Catalonia, Spain) that are candidates for day care treatment after receiving inpatient detoxification will be asked to participate in the study.

All participants will be recruited prior to CUD treatment randomization. To enter the study participants will: (a) be aged between 18 and 65 years, (b) be hospitalized for cocaine detoxification as the main substance, (c) abstinent at the time of study assessment (from the third day of hospitalization), (d) be eligible to attend day care after hospitalization. Exclusion criteria will be: (a) indication of treatment for other substance use detoxification which is not cocaine, (b) presence of severe psychopathological or neuropsychological alterations that hinder the participation in the study, (c) opioid use disorder within 1 year prior to participation (this criteria includes maintenance treatment with methadone or another opioid substance), (d) lack of Spanish or Catalan knowledge or difficulties to read or write that hampers the participation in the study, (e) non-acceptance of the study procedures, such as the signing of informed consent.

### Sample size calculation

Accepting an alpha risk of 0.05 and a beta risk of 0.2 in a two-sided test, 37 subjects are necessary in each treatment arm to recognize as statistically significant a minimum difference of 2.1 points on treatment relapse (i.e.,: Number of positive urine specimens submitted; primary outcome) between the two groups. The common deviation is assumed to be 3.1. It has been anticipated a drop-out rate of 5%. The parameters implemented to estimate the sample size are based on previous studies with similar populations (19).

### Design and settings

This is an unicentric national open-label-randomized clinical trial focused on the improvement of cocaine use disorder treatment as usual (TAU) outcomes through the implementation of a web-based CBT treatment (CBT4CBT). The study was approved by the Research Ethics Committee of the Hospital de la Santa Creu i Sant Pau, project number IIBSP-CBT4CBT-2020-1116.

**Figure 1:**
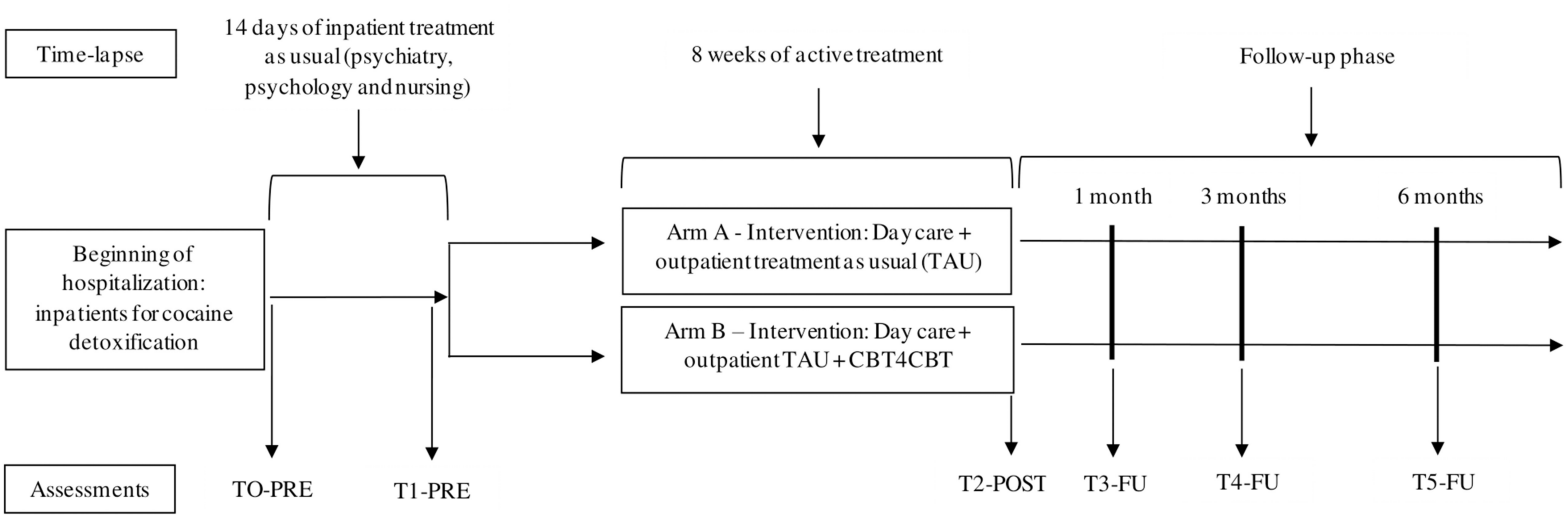
Study design.

The present RCT has two arms: arm A (TAU) and arm B (TAU + CBT4CBT). All individuals with cocaine use disorder coming to our inpatient unit for cocaine detoxification will receive treatment as usual (TAU), which will consist of a multidisciplinary approach including psychiatrists, psychologists and nurses. The hospitalization for detoxification begins with the removal of all drugs and, during hospitalization, psychiatrists adjust medication in accordance to cocaine withdrawal symptoms. Additionally, individuals also receive daily psychological interventions based on motivation for change, craving management, assertive communication, problem solving skills and relapse prevention. Nursing staff carries out necessary therapeutic actions, such as taking vital signs, administering medications, provide space for emotional venting and conducting group therapies addressing motivation for change. After a mean of 14 days from admission, inpatients are discharged and placed to either TAU (day care treatment as usual + weekly outpatient follow up) or TAU + CBT4CBT (day care treatment as usual + weekly outpatient follow up and CBT4CBT). The intervention will be discontinued in case of request by the participant. Finally, to ensure adherence to intervention, after one unjustified absence rated by the psychologist, a phone call will be done (see Figure 1).

Within the framework of the study and with the purpose of introducing the subsequent outpatient treatment, all patients in our inpatient unit for cocaine detoxification who are part of the study will also receive individual psychological CBT based treatment with motivational strategies, emotional regulation skills for mood symptoms and craving management, as explained previously. After completing the inpatient care (at discharge of inpatient care), patients will be randomly assigned to the CBT4CBT + TAU or to TAU. The randomization will be centralized through Clinapsis (http://www.clinapsis-estudis.com/) and all data will be handled through this same platform. Clinapsis is a friendly-user platform where all data from participants is stored. To ensure the transparency and the achievement of ethics procedures, the Central Clinical Research and Clinical Trials Unit of Research Institute of the Hospital de Sant Pau can access the platform to monitor the data collection and management. In addition, the two researchers who provide the psychological strategies during both the inpatient and outpatient treatment phase and carry out the assessments (NMB and APL) also have access to Clinapsis to check whether all data is completed and, subsequently, export it to the software Statistical Package for Social Sciences (SPSS) v. 26.0. Once the database is crated in SPSS, authors will manage the database removing duplicates, identifying missing values and setting up all the variables in order to conduct the statistical analyses in SPSS. Any imbalance in the number of participants in each treatment arm due to non-adherence to treatment protocol prior to starting CBT4CBT but after arm allocation, will also be centralized through Clinapsis.

### Web-based CBT intervention (CBT4CBT)

After completing the inpatient care (at discharge), the patients randomly assigned to the experimental condition will receive the TAU + CBT4CBT a web-based treatment which, as previously stated was tested on individuals with cocaine use disorder in America by the team of Carroll et al. (18). Depending on the patients' personal situation, they will have two options for receiving the treatment: they will be provided with access to the computer program in a small private room within the addictive behaviors outpatient unit or they will be provided with code access for being able to do it from home. In all instances, a psychologist will guide participants through their initial use of the CBT4CBT program and she will be available to answer questions and assist participants each time they use the program (the CBT4CBT and TAU treatments are always explained by a woman). The participants will access the program through an identification/password system to protect confidentiality.

The CBT4CBT program is intended to be user-friendly. The multimedia style of the program is based on elementary-level computer learning games, and the material is presented in a range of formats, including graphic illustrations, videotaped examples where a story with different characters with substance addictions display common risk situations and several ways to cope with them (highlighting the most functional one), verbal instructions, audio voiceovers, interactive assessments with a dichotomous response type, and practice exercises, such as decisional balances that participants must complete with their own arguments. The program consists of six skills (understanding and changing patterns of substance use, coping with craving, refusing offers of drugs, problem-solving skills, identifying and changing thoughts about drugs and improving decision making skills) organized in seven modules of 30 min duration based on a CBT manual published by the National Institute on Drug Abuse and it covers the following core concepts: (1) recognize the triggers, (2) stand up for yourself, (3) deal with craving, (4) stop and think, (5) plan don't panic, (6) go against the flow and (7) stay safe. Each participant has its own credentials to enter the platform, and once in the web-based treatment, the first module will also provide a brief explanation of how to use and navigate the program. Following completion of the first module, the participants can access the rest of the modules (see Table 1). Additional details regarding CBT4CBT can be found elsewhere (14, 19, 22).

**Table 1:** Content of the 8-week CBT4CBT program.

**Session**	**Topics covered**
Module 1: Recognize the triggers	Identify, recognize and change triggers and patterns of use
Module 2: Stand up for yourself	Assertiveness and learning the ability to say “no” when someone is offering drugs
Module 3: Deal with craving	Strategies to manage substance related craving and urges
Module 4: Stop and think	Identify distorted beliefs about drugs, as well as challenging and changing negative thoughts that are maintaining the substance use
Module 5: Plan don't panic	Improve skills and strategies to facilitate decision-making without using drugs
Module 6: Go against the flow	Improve skills to manage adverse events other than using cocaine or other substances to face problems
Module 7: Stay safe	Develop skills to prevent relapse and maintain healthy behaviors

Researchers NMB and APL monitor whether participants complete the modules of the CBT4CBT treatment and report it in a separate excel sheet. Also, the completion of the modules can be tracked entering to the section “providers” in the CBT4CBT website (http://www.cbt4cbt.com). Apart from whether the participant has completed or not the module, the researcher can check the percentage of completion of the module and the time spent on it.

## Data collection and assessment

### Assessment

There will be 6 time-point assessments: *TO-PRE:* within the inpatient unit, before starting psychological treatment but once the patients are stabilized from cocaine withdrawal; *T1-PRE:* after inpatient treatment before starting outpatient treatment (TAU or TAU + CBT4CBT); *T2-POST:* once the 8-w outpatient treatment is completed (TAU or TAU + CBT4CBT); *T3-FU: 1* month follow-up; *T4-FU:* 3 months follow-up; *T5-FU:* 6 months follow-up. Additionally, the presence of benzoylecgonine (metabolite of cocaine) in urine will be assessed weekly during the 8 w period of web-based treatment (see Table 2).

**Table 2:** Schedule of enrolment, interventions and assessments.

**Indicator**	**Enrolment (inpatient treatment)**	**End of inpatient treatment**	**TAU + CBT4CBT/TAU (weekly control)**	**Post treatment (TAU + CBT4CBT/TAU)**	**Follow-up (1, 3, 6 m)**
Inclusion/exclusion criteria	XXXX				
Informed consent signature	XXXX				
Allocation	XXXX				
Sociodemographic data collection	XXXX				
**Data about web-based CBT4CBT intervention program**					
Program satisfaction (CSQ-8)		XXXX		XXXX	
Knowledge about program content	XXXX			XXXX	
System Usability Scale (SUS)				XXXX	
User Experience Questionnaire (UEQ)				XXXX	
Usefulness, Satisfaction and Ease of use (USE) questionnaire				XXXX	
**Hospitalization data- anthropometric and biochemical variables**					
Toxicology urinalyses screen	XXXX		XXXX	XXXX	XXXX
Height	XXXX				
Weight	XXXX	XXXX		XXXX	XXXX
Cardiac frequency	XXXX	XXXX		XXXX	
Used substances (previous 30 days)	XXXX	XXXX		XXXX	XXXX
Substance use history	XXXX				
**Psychopathological status, dependence and craving**					
Beck Depression Inventory (BDI)	XXXX^**^	XXXX		XXXX	
State-Trait Anxiety Inventory (STAI)	XXXX^**^	XXXX		XXXX	
Mini International Neuropsychiatric Interview (MINI)	XXXX^**^				
Cocaine Selective Severity Assessment (CSSA)	XXXX^**^	XXXX		XXXX	
Severity of Dependence Scale (SDS)	XXXX^**^	XXXX		XXXX	
Weiss Cocaine Craving Scale (WCS)	XXXX^**^	XXXX		XXXX	XXXX
Symptom Check List Revised (SCL-90-R)	XXXX^**^	XXXX		XXXX	XXXX
**Other addictive behaviors**					
South Oaks Gambling Screen (SOGS)	XXXX^**^			XXXX	
Yale Food Addiction Scale 2 (YFAS-2)	XXXX^**^			XXXX	
DSM-5 criteria for Internet Gaming Disorder (IGD)	XXXX^**^			XXXX	
Hypersexual Behavior Inventory (HBI)	XXXX^**^			XXXX	
**Emotional regulation and personality**					
Sensitivity to Punishment and Sensitivity to Reward Questionnaire (SPSRQ)	XXXX^**^				
Difficulties in Emotion regulation Scale (DERS)	XXXX^**^	XXXX		XXXX	
Emotion Regulation Questionnaire (ERQ)	XXXX^**^	XXXX		XXXX	
UPPS-P Impulsive Behavior Scale (UPPS-P)	XXXX^**^	XXXX		XXXX	
Multidimensional Assessment of Interoceptive Awareness (MAIA)	XXXX^**^				

** Once the patient is stabilized from cocaine withdrawal (up from 48 to 72 h after hospitalization).

### Instruments

Primary outcome measures will be treatment dropout (two consecutive failures to attend and not answering the phone) and relapse (cocaine use: number of positive urine specimens submitted, percent negative urine screens, percent days of abstinence, self-reported frequency of cocaine use). Treatment dropout will be determined throughout the attendance at the appointments with the clinician (TAU or TAU + CBT4CBT), which will be carried out weekly during the 8-week period of treatment and at follow-up points. Relapse will be determined according to the participants' reports and throughout the presence of benzoylecgonine (metabolite of cocaine) in urine assessed with the Kinetic Microparticle Interaction test in a Solution (Roche Diagnostic System, Inc., Sommerville, NJ).

Secondary measures will cover sociodemographic variables and anthropometric measures (weight and high), cocaine craving, psychopathological symptoms and comorbidities, other addictive behaviors, personality traits, emotion regulation skills and CBT4CBT usability as well as treatment satisfaction.

Psychopathological symptoms and comorbid disorders will be assessed with a Clinical interview based on the DSM-5 diagnostic criteria for addictive behaviors, the MINI international neuropsychiatric interview (23), the Symptom CheckList-90 items-Revised (SCL-90-R) (24, 25), The State-Trait Anxiety Inventory (STAI) (26, 27) and the Beck's Depression Inventory (BDI) (28, 29). Craving and compulsive drug use will be measured with the following self-reported questionnaires: Severity of Dependence Scale (SDS) (30, 31), Cocaine Selective Severity Assessment (CSSA) (32, 33), Weiss Craving Scale (WCS) (34, 35). Other addictive behaviors will be measured, including gambling with the South Oaks Gambling Screen (SOGS) (36, 37), food with the Yale Food Addiction Scale 2 (YFAS) (38, 39), gaming with the DSM-5 criteria for internet gaming disorder (IGD) (40, 41) and sexuality with the Hypersexual Behavior Inventory (HBI) (42, 43).

Different facets of emotion regulation will be assessed with the following self-reported questionnaires: Difficulties in Emotion Regulation Scale (DERS) (44, 45), Emotion regulation questionnaire (ERQ) (46, 47), and Multidimensional Assessment of Interoceptive Awareness (MAIA) (48, 49). Impulsive related traits will be measured with the UPPS-P Impulsive Behavior Scale (50, 51) and the Sensitivity to Punishment and Sensitivity to Reward Questionnaire (SPSRQ) (52). Finally, treatment satisfaction will be assessed through the Client Satisfaction Questionnaire (CSQ-8) (53, 54), usability of the intervention will be assessed using the System Usability Scale (SUS) (55, 56), user experience will be assessed by the User Experience Questionnaire (UEQ) (57, 58) and usefulness, satisfaction and ease of use will be assessed with the Usefulness, Satisfaction and Ease of Use (USE) questionnaire (59, 60).

### Data analysis

Statistical programs SPSS 26.0 and Stata 15.1 for Windows will be used. The primary objectives of this study will be carried out through cox regression models. In addition, repeated measures ANOVA will also be performed to assess whether there is a time effect on the primary measures (treatment dropout and/or relapse) as well as secondary (craving, psychopathological state, recent cocaine use emotion regulation scores, trait impulsivity, other addictive behaviors) measures. In addition, these scores will be compared between the two treatment groups using a mixed model of repeated measures. All statistical tests will be two-tailed and will be considered significant if p <0.05. Also, for the characterization of the differences between men and women group comparisons will be conducted by means of chi-square tests (categorical variables) and *t*-tests (continuous variables). Finally, generalized linear models will be implemented to assess the psychopathological factors associated with treatment response. The need for the analyses to be adjusted for potential confounding variables that could introduce a bias in the results will be considered (e.g.,: age, psychopathology, among others). Especially if the study groups present an imbalance with confounding variables reported in previous literature.

## Discussion

CBT4CBT is a CBT web-based treatment which concomitant to TAU has shown to improve current treatments in multiple US-based studies (18–20). However, up to date it has never been implemented in Spain.

This study aims to improve current evidence-based treatments for severe CUD (that requires inpatient treatment) through the implementation of a more intensive and extensive treatment which is grounded on a web-based CBT approach (CBT4CBT). Additionally, predictive factors for treatment outcome will be assessed. The main strength and contribution of this study will be the enhancement of CUD treatment, using a concomitant web-based treatment approach to TAU. CBT4CBT has the potential to optimize current treatments by facilitating daily access to it when needed. This study also explores different secondary measures (i.e., sociodemographic, cocaine use related variables, craving, personality traits, as well as comorbid psychopathology and other addictive behaviors) that can help to better understand predictive factors of treatment outcomes at two different clinical settings (i.e., inpatients treatment and day care treatment + CBT4CBT) and at 6 different time points (end of each treatment setting and at three different follow-up points−1, 3, and 6 months after treatment-). This can lead to the possibility of targeting specific characteristics in a population of severe CUD and to individualize treatments accordingly for aiming to a better outcome. Furthermore, this study will take into account a gender perspective and explore gender differences in predictive factors for treatment outcome and the efficacy of CBT4CBT.

The potential limitations of this study are the exclusion of patients with opioid use disorder or any other severe condition that can interfere in the participation or assessment of the study. Additionally, dropout rates are considered as treatment outcome although participants can dropout before starting CBT4CBT or TAU after arm allocation which would limit the power of the analysis. Finally, some patients will not be able to join the study if they live in a different area or attend to another treatment than day care. This is required for enabling the implementation of the full treatment and may be strength for homogeneity but it can also limit the generalization of results.

## Ethics statement

The study was approved by the Research Ethics Committee of the Hospital de la Santa Creu i Sant Pau, project number IIBSP-CBT4CBT-2020-1116. The study will be carried out in accordance with the Declaration of Helsinki and Good Clinical Practices ethical principles. All participants will be informed about the study and they will have to provide informed consent for participation before their inclusion. This study does not pose any risk to the participants. Central Clinical Research and Clinical Trials Unit of Research Institute of the Hospital de Sant Pau will monitor through periodic visits the correct progression of the project, notifying protocol deviations. They will be aware of every single step of the project.

## Author contributions

NM-B designed the study with the collaboration of JT and SD-S. NM-B, JT, SD-S, FB, and MM were involved in the set-up of the study in the clinical settings. NM-B and AP-L drafted the manuscript. JT, SD-S, FB, and MM critically reviewed the manuscript. All authors read and approved the final version of the manuscript.
